# Supplementary material for: Nontrivial band geometry in an optically active system
Source: Nat Commun. 2021 Jan 29;12:689. doi: 10.1038/s41467-020-20845-2 (PMC7846789; doi:10.1038/s41467-020-20845-2)
Supplement: Supplementary file 2 — Supplementary Information [file 41467_2020_20845_MOESM2_ESM.pdf]

Supplementary Information
Nontrivial band geometry in an optically active system

Ren et al

Supplementary Figures

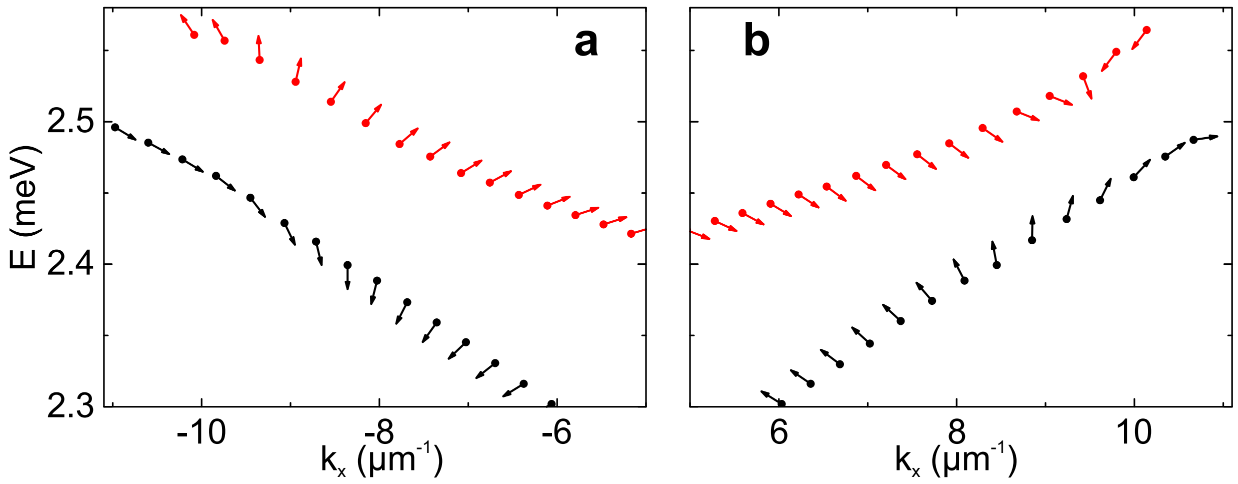

Supplementary Figure 1: **Eigenstates at the anticrossing.** Experimentally extracted dispersion of the upper and lower bands (E_0 and E_1 from Fig. 2(e)) around the two anticrossing points (a and b). The experimentally extracted pseudospin of the eigenstates is shown with black arrows (Stokes vector components (S_1, S_3)). Opposite circular polarization is observed for opposite wave vectors, while the in-plane component demonstrates convergent and divergent textures.

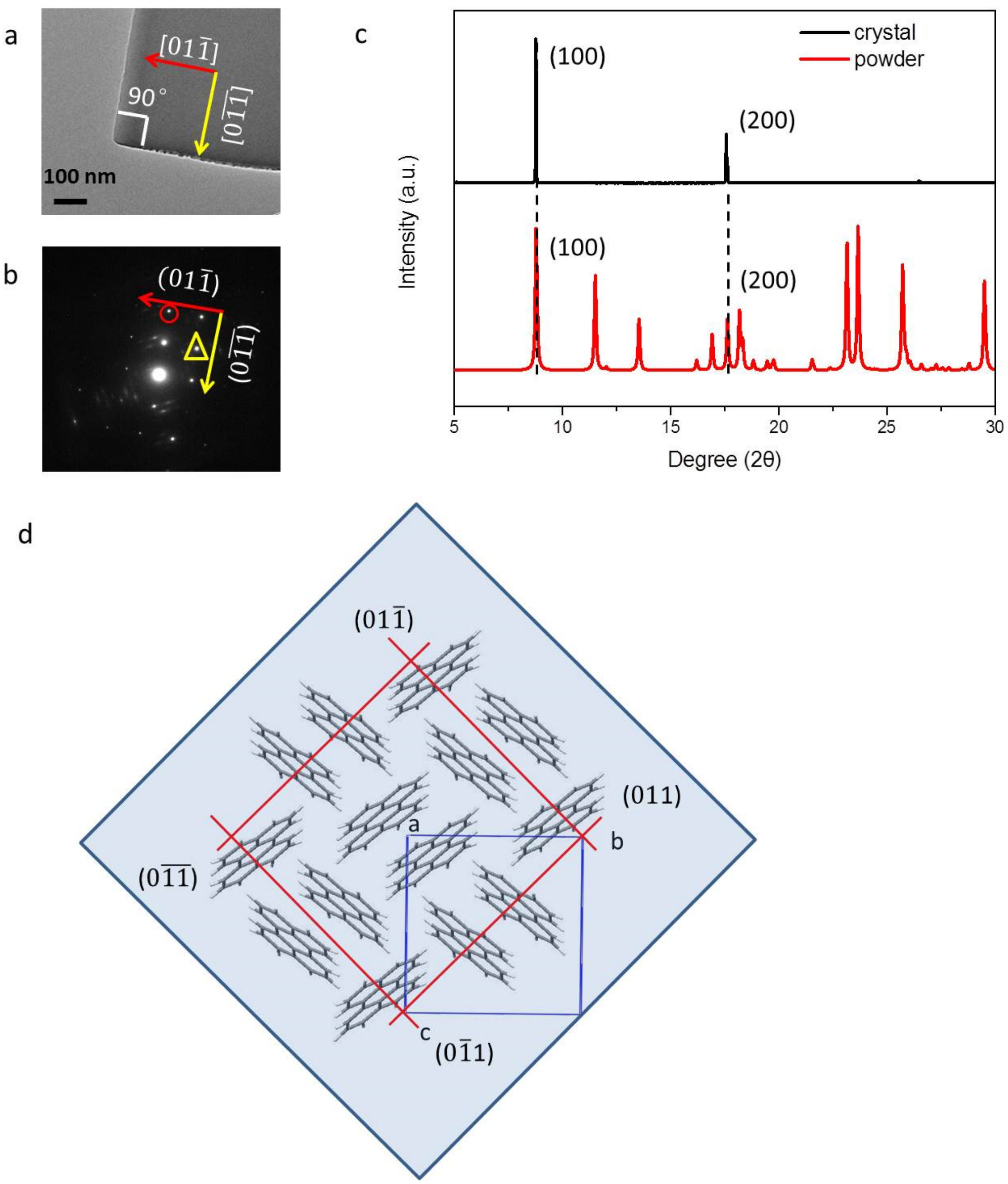

Supplementary Figure 2: **Material structure.** A and B: TEM image (A) and the corresponding SAED pattern (B) of the perylene microcrystal; C: XRD pattern of the perylene microcrystal (upper panel, black line) and the perylene powder used to fabricate the microcrystal (lower panel, red line); D: The simulated molecule arrangement of perylene from top view. The straight colored lines label the crystal faces (red line) and the unit cell (blue line).

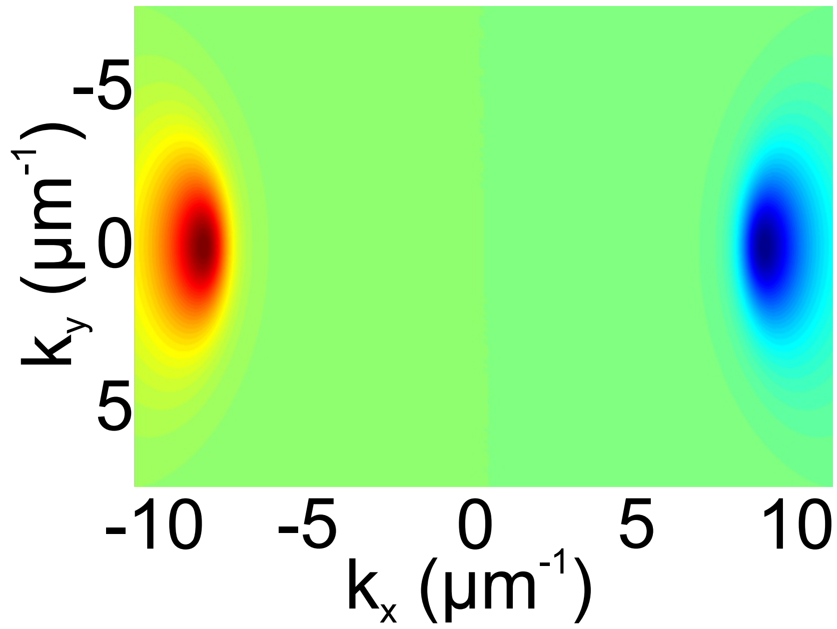

Supplementary Figure 3: **Berry curvature distribution.** Calculated Berry curvature distribution based on a 4×4 effective Hamiltonian including linear and circular polarization splittings, and a Rabi splitting term (140 meV), coupling the excitons and the photons.

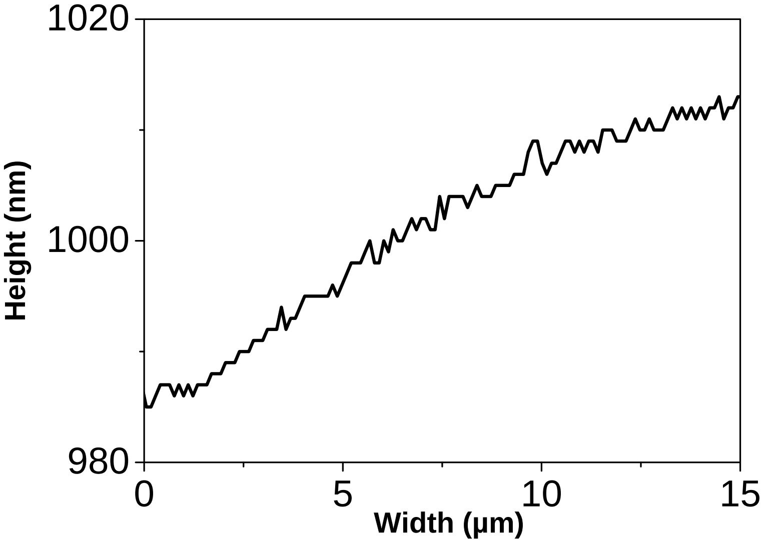

Supplementary Figure 4: **Cavity thickness variation.** Experimentally measured cavity thickness exhibiting a gradient of ≈ 1.3 nm/μm for the whole structure.

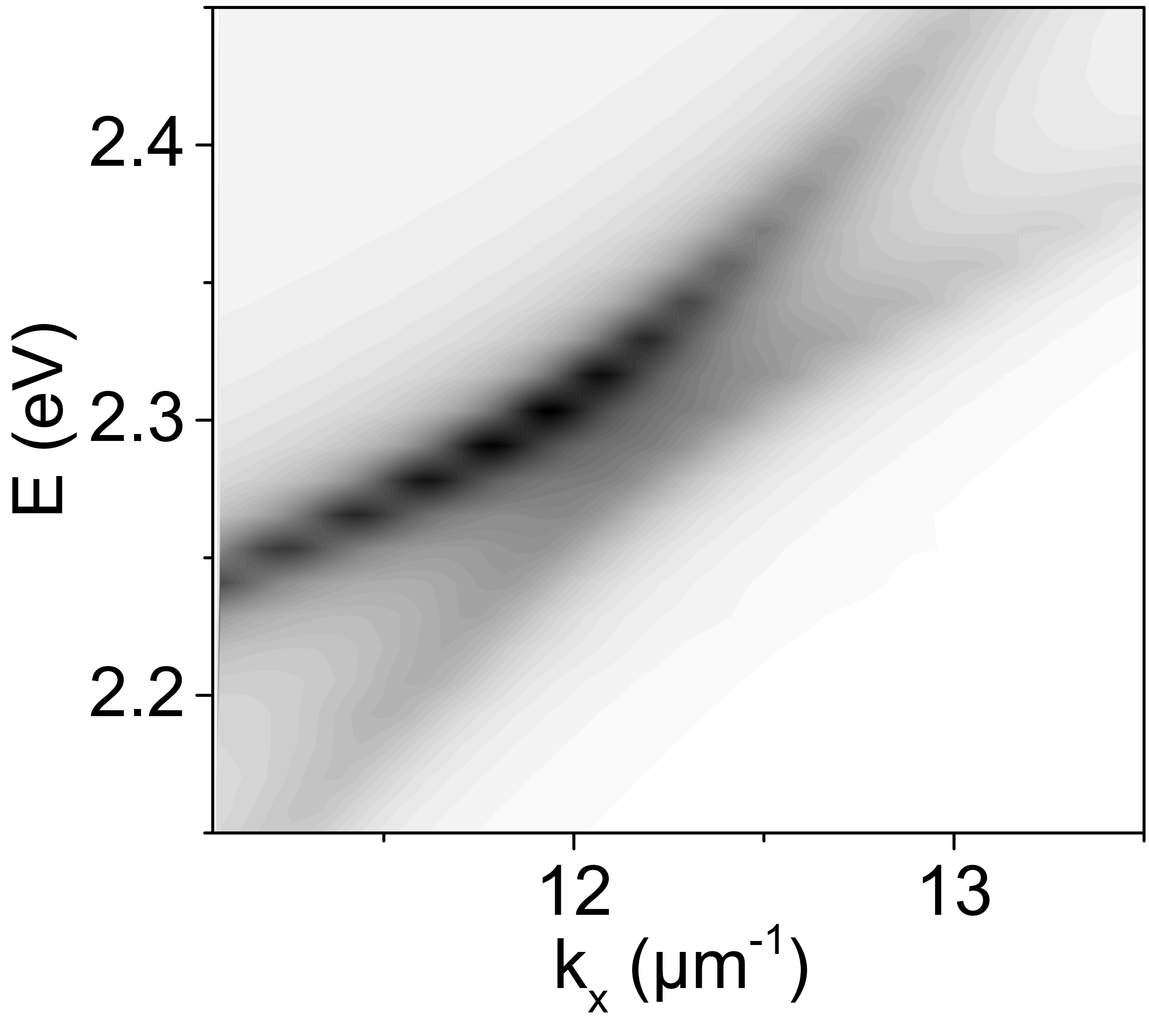

Supplementary Figure 5: **Anticrossing in numerical simulations.** Numerical simulations with COMSOL, showing the transmissivity as a function of energy and wave vector. An anticrossing of the opposite-parity branches appears due to the emergent OA.

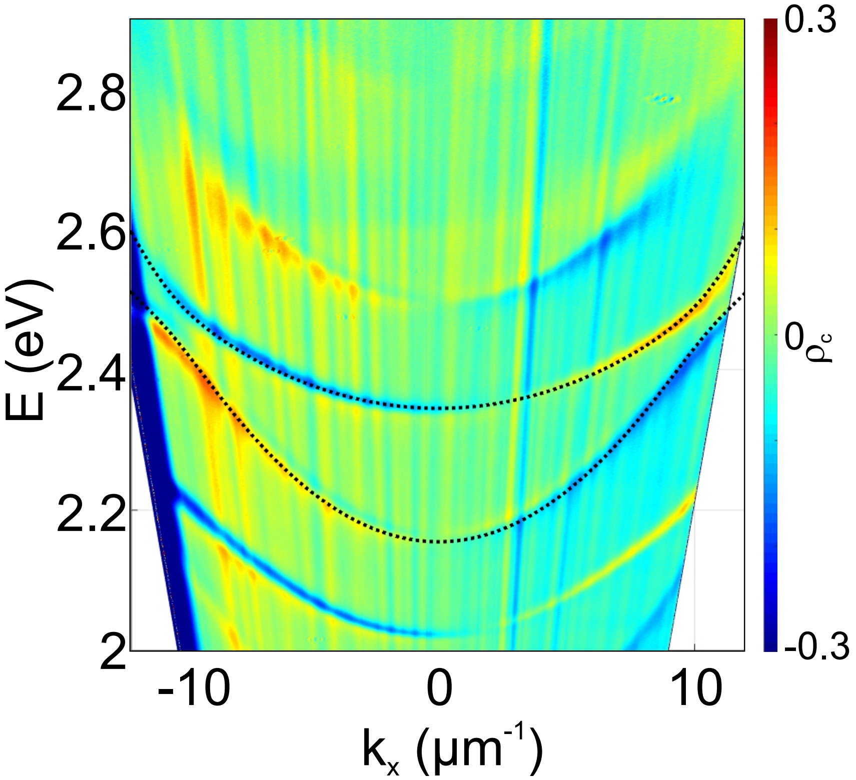

Supplementary Figure 6: **Circular polarization degree of the reflectivity.** Experimentally measured circular polarization degree (conditions as in Fig. 2(e) of the main text). The theoretically calculated dispersions are shown as dotted lines. A clear antisymmetric behavior is visible for all branches.

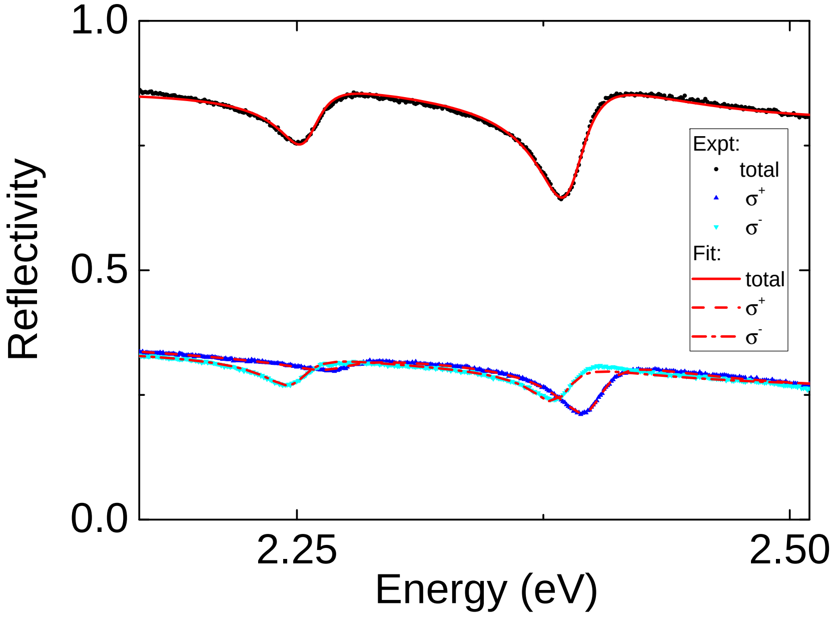

Supplementary Figure 7: **Reflectivity spectrum.** A typical reflectivity spectrum for a single point of the reciprocal space (experimentally measured points and theoretically calculated lines) showing the total reflectivity (black circles, red solid line) and reflectivity measured in circular polarization (blue and cyan triangles, red dashed and dash-dotted lines). The theoretical fit is used to extract the mode positions and their S_3 value.
